# Generalised Joint Hypermobility in Caucasian Girls with Idiopathic Scoliosis: Relation with Age, Curve Size, and Curve Pattern

**DOI:** 10.1155/2014/370134

**Published:** 2014-01-16

**Authors:** Dariusz Czaprowski

**Affiliations:** Department of Physiotherapy, Józef Rusiecki University College in Olsztyn, Bydgoska 33, 10-243 Olsztyn, Poland

## Abstract

The aim of the study was to assess the prevalence of generalised joint hypermobility (GJH) in 155 girls with idiopathic scoliosis (IS) (age 9–18 years, mean 13.8 ± 2.3). The control group included 201 healthy girls. The presence of GJH was assessed with Beighton (B) test. GJH was diagnosed in 23.2% of IS girls and in 13.4% of controls (*P* = 0.02). The prevalence of GJH was significantly (*P* = 0.01) lower in IS girls aged 16–18 years in comparison with younger individuals. There was no difference regarding GJH occurrence between girls with mild (11–24°), moderate (25–40°), and severe scoliosis (>40°) (*P* = 0.78), between girls with single thoracic, single lumbar, and double curve scoliosis (*P* = 0.59), and between girls with thoracic scoliosis length ≤7 and >7 vertebrae (*P* = 0.25). No correlation between the number of points in B and the Cobb angle (*P* = 0.93), as well as between the number of points in B and the number of the vertebrae within thoracic scoliosis (*P* = 0.63), was noticed. GJH appeared more often in IS girls than in healthy controls. Its prevalence decreased with age. No relation between GJH prevalence and curve size, curve pattern, or scoliosis length was found.

## 1. Introduction

Idiopathic scoliosis (IS) has been described as a torsional deformity of the spine, which combines a translation and rotation of a variable number of vertebrae, changing the 3D geometry of the spine [1]. Although the cause of idiopathic scoliosis remains unknown, different theories concerning its etiology have been proposed [2, 3]. Some of them draw attention to abnormalities within muscle, bone, ligaments, and disc [3]. These theories are based on observations that conditions such as Marfan syndrome or fibrous dysplasia of bone are associated with the development of scoliosis. The collagen fiber defects of Marfan syndrome and osteogenesis imperfecta have also led researchers to investigate the connective tissues in idiopathic scoliosis [3]. The term “dangerous triad” introduced by Tanchev et al. might confirm a vital role of soft tissues in the etiology of scoliosis as well [4]. According to these authors, generalised joint hypermobility along with delayed maturity and asymmetric spinal loading are major factors influencing the development of idiopathic scoliosis.

According to actual SOSORT Consensus, the following factors have been suggested as possible determinants of a higher risk of scoliosis progression: positive family history, flattening of physiological thoracic kyphosis, angle of trunk rotation exceeding 10°, and growth spurt [1]. The joint hypermobility might influence the risk of progression as well [1].

In accordance with the guidelines provided by SOSORT for children with mild and moderate idiopathic scoliosis, conservative treatment is recommended [1]. This consists of observation, physiotherapeutic specific exercises, special inpatient rehabilitation, and bracing [1]. The main aim of exercises is to improve spinal stability to decrease the risk of scoliosis progression [1]. However, some of the physiotherapeutic methods applied to treat scoliotic children also use the exercises aimed at increasing spinal mobility to achieve a potential curve correction [5–8].

Generalised joint hypermobility (GJH) is diagnosed when the mobility of small and large joints is increased in relation to standard mobility for any given age, gender, and race and after excluding systemic diseases [9]. GJH occurs more frequently in younger children, girls as well as Asians and Africans [10, 11].

Muscle strengthening as well as proprioception, stability and balance improvement are recommended in management of children with joint hypermobility syndrome [10, 12–14]. In turn, exercises aimed at increasing joint mobility should not be used as they may be harmful [13]. Therefore, disregarding the probability of hypermobility syndrome occurring in children when planning the physical therapy might lead to nonoptimal management [12].

A limited number of studies evaluating the prevalence of GJH in IS patients have been carried out so far. Their results suggest that joint hypermobility appears in children with idiopathic scoliosis more frequently than in healthy individuals of the same age [15, 16]. However, these studies were either conducted in small study groups [16] or were based on the mobility of a thumb only [15]. On the other hand, Mattson et al. analysed joint flexibility in 51 girls with untreated mild idiopathic scoliosis and their conclusion is that there is no difference regarding joint flexibility between scoliotic girls and normal controls [17]. Thus, it is essential to make an observation using tests assessing the mobility of a number of joints and covering a large group of individuals with idiopathic scoliosis in order to verify the prevalence of generalised joint hypermobility in idiopathic scoliosis patients. The obtained results might improve the planning of conservative treatment of IS patients.

The aim of the study was to assess the prevalence of generalised joint hypermobility in girls with idiopathic scoliosis and to assess the relationship between the GJH occurrence and the age of IS patients, the angle of curvature, the number of curvatures, and the localization and the length of scoliosis.

## 2. Material and Methods

The study included 155 girls aged 9–18 years (mean 13.8 ± 2.3) with idiopathic scoliosis. The criteria for inclusion to the study group were as follows: diagnosis of idiopathic scoliosis on anteroposterior radiogram in accordance with Scoliosis Research Society criteria (Cobb angle >10° with rotation), single right thoracic, single left lumbar, or double major thoracolumbar scoliosis, absence of systemic diseases related to joint hypermobility (Ehlers-Danlos, Marfan, Larsen), and absence of musculoskeletal pain throughout the previous 6 months. Cobb angle range was 11°–65°, mean 28.2° ± 12.1, comprising 44 single thoracic, 35 single lumbar, and 76 double curve scoliosis. According to Cobb angle, there were 74 mild (11–24°), 57 moderate (25–40°), and 24 severe (>40°) curves. The average number of vertebrae forming single-curve thoracic scoliosis was 7.4 vertebrae (range 4–11). The recruitment of the girls to the control group took place during the presentations for parents and their daughters. The presentations were given in 8 randomly selected schools. The information about the study was placed on notice boards and school websites with the school master's consent. 290 parents and their daughters participated in the meetings. Finally, the control group included 201 girls, who met the following criteria: age range 9–18, less than 5° of angle of trunk rotation as measured with Bunnell scoliometer, absence of systemic diseases, and absence of musculoskeletal pain in the previous 6 months. Prior to the study, written consent of parents was obtained in which they allowed their children to participate in the study. The local Ethical Commission granted permission for this research.

The presence of GJH was assessed with the nine-point Beighton test (hyperextension of the MCP joint of the fifth finger >90°; abduction of the thumb to the forearm; elbow hyperextension >10°; knee hyperextension >10°; and touching the floor with the palms of the hands during trunk forward bend performed in a standing position) [18, 19], using the cut-off ≥5 points [20]. In order to assess the range of joint mobility, a set of goniometers was used (Baseline, USA).

The prevalence of GJH was compared between the study and control group. The relation of GJH occurrence to the following parameters was assessed: (1) the age of patients—the comparison of the GJH prevalence between girls aged 9–12 years, 13–15 years, and 16–18 years; (2) correlation between the number of points in the Beighton test and the age, (3) the Cobb angle value—comparison between mild, moderate, and severe scoliosis; (4) the Cobb angle value—correlation between the number of points in the Beighton test and the Cobb angle; (5) number and localization of curvatures—single thoracic versus single lumbar versus double curve scoliosis; (6) number of vertebrae within the single thoracic scoliosis—below versus above the mean of 7.4 vertebrae; (7) number of vertebrae within the single thoracic scoliosis—correlation between the number of points in the Beighton test and the number of vertebrae within the single thoracic scoliosis.

Statistical analysis was performed with Statistica 7.1 (StatSoft, Poland). The Shapiro-Wilk test was applied to the analysis of the normal distribution. The *U* Mann-Whitney, Chi^2^, and Kruskal-Wallis tests as well as Spearman correlation rank were conducted. The value *P* = 0.05 was adopted as the level of significance.

## 3. Results

Girls from the study group were comparable to girls from the control group with respect to age (*P* = 0.2), height (*P* = 0.2), weight (*P* = 0.5), and BMI (*P* = 0.5) (Table 1).

There were no significant age differences (*P* = 0.1) between girls with mild, moderate, and severe scoliosis (median (QR), 13.5 (2.4) for mild versus 13.9 (2.2) for moderate versus 14.6 (2.3) for severe scoliosis). No significant differences were found regarding Cobb angle (*P* = 0.22) and the age (*P* = 0.24) between girls with single thoracic, single lumbar, and double curve scoliosis (median (QR), 31.5° (15.5) versus 24.0° (17.0) versus 30.0° (17.0) for Cobb angle and 13.5 (3.5) versus 15.0 (2.0) versus 14.0 (3.0) for age). There were no significant differences regarding Cobb angle (*P* = 0.85) and the age (*P* = 0.14) between girls with shorter (≤7 of vertebrae within curvature) and longer (>7) thoracic scoliosis (median (QR) 33.0° (13.0) versus 30.0° (18.0) for Cobb angle and 14.0 (4.0) versus 13.0 (4.0) for age).

GJH was diagnosed in 23.2% of IS girls, whilst in the control group it was diagnosed in 13.4% (*P* = 0.02) (Table 2). The prevalence of GJH was significantly (*P* = 0.01) lower in IS girls aged 16–18 years in comparison with younger individuals (9–15 years) (Table 3). There was no difference regarding GJH occurrence between girls with mild, moderate, and severe scoliosis (*P* = 0.78) (Table 4). No significant differences in GJH prevalence were observed between girls with single thoracic, single lumbar, and double curve scoliosis (*P* = 0.59) (Table 5). There was no difference regarding the prevalence of GJH between girls with thoracic scoliosis length below or equal and above 7 vertebrae (*P* = 0.25) (Table 6). The significant correlation between the number of points obtained in the Beighton test and the age of IS girls (*P* = 0.03) was observed. No significant correlation between the number of points in the Beighton test and the Cobb angle (*P* = 0.93) as well as the number of the vertebrae within thoracic scoliosis (*P* = 0.63) was noticed (Table 7).

## 4. Discussion

The aim of this study was to assess the frequency of GJH occurrence among girls with IS and to evaluate the relationship between GJH occurrence and the age of subjects as well as the radiological parameters of scoliosis. The study showed that GJH occurs more frequently in scoliotic girls in comparison with healthy peers (23.2% versus 13.4%, *P* = 0.02). These results are confirmed by the observations made by Binns [15] and Czaprowski et al. [16]. Binns found that the girls with adolescent idiopathic scoliosis demonstrate greater laxity [15]. However, it should be noted that Binns evaluated joint laxity merely by measuring the distance from the thumb to the forearm. Moreover, his observations concerned Asians only and therefore the direct comparison of the results is impossible. Binns suggested that those patients who demonstrate hyperlaxity may be more likely to progress to scoliotic deformity presumably because of reduced spinal integrity. Although this conclusion is interesting, it seems that it requires further research. Czaprowski et al. concluded that joint hypermobility occurs significantly more often in both boys and girls with IS in comparison with the control group (50.8% versus 21.0% and 56.1% versus 44.4% for girls and boys, resp.) [16]. However, the authors made observations on relatively small groups (59 scoliotic girls and 11 boys as well as 33 healthy girls and 25 healthy boys). The present study shows the analysis of GJH occurrence in 155 girls with idiopathic scoliosis. To the best of the author knowledge, it is the largest group of IS girls analysed with respect to GJH occurrence. Therefore, it forms the basis for more thorough evaluation of the relationship between GJH occurrence and parameters of IS.

The criterion for diagnosis assumed by Czaprowski et al. was obtaining ≥4 points in the 9-point Beighton test without differentiating the threshold value in terms of gender [16]. Hakim et al. indicate that obtaining at least 4 points should be assumed as the basis of diagnosis in boys whereas in girls obtaining ≥5 points confirms the diagnosis of GJH [20]. Therefore, such a threshold value was assumed in the present study. I believe that adopting this value enabled me to properly evaluate the frequency of GJH occurrence in girls with IS. However, direct comparison of the results of the present study with the results of other authors is made difficult.

Generalised joint hypermobility presents the natural tendency to decrease its prevalence with age [6, 18, 21]. The study confirmed that the GJH occurs significantly more rarely in girls aged 16–18 years in comparison with younger girls (5.6% versus 25.6%, for girls aged 13–15 years and versus 34.2%, for girls aged 9–12 years, *P* = 0.01). The number of points obtained in the Beighton test also decreased with age (*P* = 0.03).

The present study did not indicate a significant relationship between the frequency of GJH occurrence and the angle of scoliotic curvature. The GJH was recognised in 21.6% of mild, 26.3% moderate, and 20.8% severe scoliosis, and there was no significant difference between groups (*P* = 0.78). The correlation between the number of points obtained in the Beighton test and the Cobb angle was also insignificant (*P* = 0.93).

In the presented study, while analyzing the relationship between GJH prevalence and scoliosis length, only single thoracic scoliosis was taken into consideration. It resulted from the fact that I wished to avoid the potential influence of the number of curvatures and the curve localization on the obtained results. The conducted analysis did not reveal a significant relationship between the number of points in the Beighton test and the number of the vertebrae within thoracic scoliosis (*P* = 0.63). However, it proves interesting that GJH was observed in 25.0% of girls with shorter curvature scoliosis in comparison with 10% of girls with longer scoliosis. Although this difference was not significant (*P* = 0.25), it seems essential to carry out further observations investigating whether the lack of a difference is merely the effect of the phenomenon of statistics or the length of scoliosis indeed does not bear any relation to the occurrence of GJH.

It is important to distinguish generalised joint hypermobility, which describes the often asymptomatic increased range of joint or spinal movement, from hypermobility syndrome, its symptomatic counterpart [10]. However, the borderline between constitutional generalised joint hypermobility versus pathological skin and joint laxity is not always easily defined [16]. Nevertheless, in everyday practice, the clinical examination remains the means to assess both GJH and pathological soft tissue laxity. It is worth noting that the latter is expressed within the skin; the assessment of the skin fold and skin laxity is an important part of the examination.

The majority of the papers describing the increased joint mobility concentrate on joint hypermobility syndrome [10–14]. In turn, there is a paucity of publications on the management of generalised joint hypermobility. Therefore, in the present study the management recommendations for joint hypermobility syndrome (JH) were used. Although the aforementioned are two different notions, the character of changes in the mobility of joints is comparable and the difference between the two concerns the occurrence of arthralgia in subjects with JH [10]. Therefore, I am convinced that adopting therapeutic recommendations for joint hypermobility syndrome in the management of GJH subjects is reasonable. Nevertheless, further research verifying this assumption should be undertaken.

The aims of the integrated physical therapy programme concentrating on JH subjects are (1) improving spinal posture by developing core stability, (2) enhancing joint stability by encouraging joint-stabilizing exercises, (3) improving joint proprioception by suitable exercises, (4) avoiding resting in harmful end-of-range postures, (5) using pacing, coping, and other behavioural strategies in severe or widespread chronic pain, (6) reversing deconditioning and enhancing fitness by aerobic exercises, (7) invoking self-management, and (8) reeducating the gait [10, 11, 14]. Stretching tight muscles is not recommended in individuals with JH since it might be harmful to them [12, 13]. It might be confirmed by the results of the study conducted by Howell who observed the increase in back pain in individuals with excessive spinal mobility who participated in a stretching programme [22]. Admittedly, the increase in the activity of some muscles (e.g., hamstrings) is observed in individuals with GJH yet it is accounted for by the phenomenon of compensation aimed at increasing the stability of unstable joints [23].

Physical therapy is recommended as the first step to treat mild idiopathic scoliosis to prevent/limit progression of the deformity and bracing [1]. In moderate scoliosis the aim of physiotherapy is to enhance the effect of brace and counteract its side-effects [24]. Along with autocorrection in 3D, training in activities of daily living, and patient education, physical therapy should be based on stabilizing the corrected posture [1]. It stems from the suggestion that good spinal stability could neutralise postural deficits and halt the progression of initial scoliosis [2].

In the description of methods widely applied in the treatment of IS patients and recommended by SOSORT as physiotherapeutic specific exercises, the author of the presented study found no information whether the probability of joint hypermobility occurrence is taken into consideration during diagnosis and afterwards in planning physical therapy [5, 8, 25–27]. The above-mentioned methods emphasise the particular significance of performing exercises which aim at improving spinal stability [5, 8, 26, 27]. A number of them also focus on such elements of therapy as: (1) symmetric mobilizing exercises to improve lordosing mobility of the lumbar spine and kyphosing mobility of thoracic spine [8], (2) mobilization exercises [26], and (3) exercises and manual techniques aimed at removing muscle contractures and releasing myofascial structures (e.g., plantar fascia, short head of the biceps muscle of the thigh or sacrotuberous ligament) [5]. Moreover, as far as other methods which are disregarded by SOSORT are concerned, the author did not find any information on whether the diagnosis of GJH is taken into account while selecting exercises [6, 7]. In these methods, exercises which are designed to increase muscle flexibility and joint as well as spinal mobility [7] or exercises aimed at removing contractures in the region of hips, pelvis, and spine [6] serve a significant role.

It is also worth noting that, in the recently published literature reviews regarding the efficiency of physical therapy in the treatment of IS patients, none of the authors of the studies stated whether the occurrence of GJH was verified prior to performing exercises [28, 29]. It might indicate that the exercises were not diversified depending on the diagnosis of GJH. Moreover, during the analysis of the literature, the author of the presented study did not encounter any publications in which the authors would indicate that the generalised joint hypermobility occurrence was taken into account prior to the evaluation of the exercises efficacy.

Tanchev et al. observed a greater incidence of IS in girls practising rhythmic gymnastics [4]. According to the authors, it indicates the important etiologic role of a “dangerous triad” (asymmetric spinal loading, delayed maturity, and generalised joint laxity). Performing exercises aimed at increasing joint mobility is typical for gymnastics training [4]. Thus, perhaps such exercises ought not to be performed by children with IS.

The aim of the present study was not to discuss the therapeutic strategy suggested in particular methods but rather to draw attention to the lack of diverse programmes of physical therapy for IS patients depending on the presence or absence of GJH. Murray emphasises that joint hypermobility is often “missed” during diagnosis process because it is not examined for in situation where the exclusion of inflammatory or destructive rheumatic conditions is the expectation of patients and physician alike [11]. Therefore, a complete musculoskeletal examination should include an assessment of specific joint and overall range of motion to detect hypermobile individuals [11]. Russek also claims that physical therapists' task is to recognise joint hypermobility and design exercises taking into account the occurrence of JH [13].

Considering the prevalence of GJH in girls with idiopathic scoliosis as well as the recommendations for exercises for children with joint hypermobility, it seems that a complete musculoskeletal examination should include specific tests (e.g., Beighton test) to detect hypermobile individuals, especially when physical therapy affecting joint mobility is planned. There is a need for further research verifying the assumption that taking into consideration the occurrence of GJH while planning physical therapy of IS patients might influence the result of treatment.

## 5. Conclusions

Generalised joint hypermobility appeared more often in girls with idiopathic scoliosis than in healthy controls. Its prevalence decreased with age. No relation between joint hypermobility prevalence and curve size, curve pattern, or number of vertebrae within curvature was found.

## Figures and Tables

**Table 1:** Parameters of the study and the control group.

	Study group *n* = 155		Control group *n* = 201		*P *
	Mean (SD)	Median (QR)	Mean (SD)	Median (QR)	
Age (years)	13.8 (2.3)	14.0 (3.0)	14.1 (2.5)	14.0 (4.0)	0.2
Height (m)	1.6 (0.1)	1.6 (0.1)	1.6 (0.1)	1.6 (0.2)	0.2
Weight (kg)	48.9 (10.3)	49.0 (13.0)	47.7 (11.7)	49.0 (18.6)	0.5
BMI (kg m^−2^)	18.9 (2.9)	18.7 (3.2)	19.1 (3.1)	18.8 (3.6)	0.5

**Table 2:** The prevalence of generalised joint hypermobility (GJH) in girls from the study and the control group.

	GJH present—*n* (%)	GJH absent—*n* (%)
Study group *n* = 155 (100%)	36 (23.2)	119 (76.8)
Control group *n* = 201 (100%)	27 (13.4)	174 (86.6)
*P *	**0.02**	

Significant difference marked in bold.

**Table 3:** The prevalence of generalised joint hypermobility (GJH) in girls with idiopathic scoliosis aged 9–12, 13–15, and 16–18 years.

	GJH present—*n* (%)	GJH absent—*n* (%)
9–12 years (*n* = 41)	14 (34.2)	27 (65.8)
13–15 years (*n* = 78)	20 (25.6)	58 (74.4)
16–18 years (*n* = 36)	2 (5.6)	34 (94.4)
*P *	**0.01**	

Significant difference marked in bold.

**Table 4:** The prevalence of generalised joint hypermobility (GJH) in girls with mild, moderate, and severe scoliosis.

	GJH present—*n* (%)	GJH absent—*n* (%)
11–24° (*n* = 74)	16 (21.6)	58 (78.4)
25–40° (*n* = 57)	15 (26.3)	42 (73.7)
>40° (*n* = 24)	5 (20.8)	19 (79.2)
*P *	0.78	

**Table 5:** The prevalence of generalised joint hypermobility (GJH) in girls with single thoracic (*T*), single lumbar (*L*), and double curve (*T* + *L*) scoliosis.

	GJH present—*n* (%)	GJH absent—*n* (%)
*T *(*n* = 44)	9 (20.5)	35 (79.5)
*L *(*n* = 35)	7 (20.0)	28 (80.0)
*T* + *L *(*n* = 76)	18 (23.7)	58 (76.3)
*P *	0.59	

**Table 6:** The prevalence of generalised joint hypermobility (GJH) in girls with thoracic scoliosis length below or equal and above 7 vertebrae.

	GJH present—*n* (%)	GJH absent—*n* (%)
≤7 (*n* = 24)	6 (25.0)	18 (75.0)
>7 (*n* = 20)	2 (10.0)	18 (90.0)
*P *	0.25	

**Table 7:** The correlation between number of points in the nine-point Beighton test and age (1), Cobb angle (2), and number of vertebrae within the single thoracic scoliosis (3).

	Beighton score	
	*R * Spearman	*P *
1	−0.171	**0.03**
2	−0.007	0.93
3	−0.039	0.63

Significant correlation marked in bold.
